# Subjective household poverty as a moderator for the association between employment precariousness and mental health across five european welfare state types

**DOI:** 10.1016/j.ssmph.2024.101696

**Published:** 2024-07-02

**Authors:** Ceciel Pauls, Maria Fleischmann, Michel Klein, Stef Bouwhuis, Judith E. Bosmans

**Affiliations:** a Department of Health Sciences, Vrije Universiteit Amsterdam, Netherlands; b Hogeschool Rotterdam, Lectoraar Verloskunde en Geboortezorg, Netherlands; c Department of Computer Science, Vrije Universiteit Amsterdam, Netherlands; d Department of Sociology, Vrije Universiteit Amsterdam, Netherlands

**Keywords:** Precarious employment, Precarious work, Welfare state regime, Income insecurity, Mental health problems, Mental well-being, Europe

## Abstract

**Objectives:**

To create better understanding of the mechanisms underlying the association between employment precariousness (EP) and mental health by considering household poverty as a moderator while stratifying for gender across welfare state types (WSTs): Scandinavian, South European, Central- and East European, Bismarckian and Anglo-Saxon.

**Methods:**

Data from the sixth wave of the European Working Conditions Survey (N = 18,725) was used. The Employment Precariousness Scale was used to assess EP on a continuous scale. Mental health was measured using the WHO-5 Well-Being Index. A binary variable for subjective household poverty was created. We estimated gender-stratified, multi-level models with a random intercept at country-level for the association between EP and mental health, with an interaction term between EP and subjective household poverty, for each WST separately. Models were adjusted for age, education, having a partner and having children under age 18 in the household.

**Results:**

In all WSTs, among men as well as women, we found a negative relation between EP and mental health. Among women, this relation was not moderated by household poverty. Among men in the Anglo-Saxon WST, the negative relation between EP and mental health was stronger among employees that reported household poverty compared to those who did not report household poverty.

**Conclusions:**

Evidence of a moderating effect of household poverty on the association between EP and mental health was only found amongst men in the Anglo-Saxon WSTs and the combined full sample. Other factors that might affect the association between EP and mental health should be investigated.

## Highlights

• Employment precariousness (EP) is negatively associated with mental health outcomes.
• Subjective household poverty (SHP) may affect the strength of the association.
• This effect differs for men and women in different welfare state types.
• SHP moderates the association between EP and mental health amongst Anglo-Saxon men.
• Amongst women, household poverty did not affect the strength of the association.

## 1 Introduction

After World War II, full-time employment with set working times became the norm (Quinlan et al., 2001) and most employees became protected by rights and legislation (Siebert, 1997; Standing, 1989). In addition, in many European countries, a welfare state was established or expanded (Korpi, 2003) by providing a variety of employment rights and protective laws to employees (Standing, 1989). From the mid-1970's onwards, however, the labour market underwent drastic changes (Artner & Soreg, 2018; Duménil & Lévy, 2005; Quinlan et al., 2001). Increased competition on the international market (Gutiérrez-Barbarrusa, 2016; Standing, 1989; Standing, 1997; Standing, 2009), inflation (Duménil & Lévy, 2005; Standing, 1989) and surging unemployment rates (Duménil & Lévy, 2005) undermined the legitimacy of the current system (Standing, 1997) as neoliberal ideas, which emphasize the role of the market and the reduction of governmental protection schemes, began to gain traction (Kalleberg, 2009). These developments inspired governments in many countries to deregulate their labour markets (Kalleberg, 2009) and employers to aim for more non-standard working relationships with their employees. This ultimately led to an increase in precarious forms of employment (Kalleberg, 2013).

There is no universally recognized definition of employment precariousness (EP) (Bodin et al., 2020), and authors thus use a variety of definitions that may encompass dimensions such as insecurity related to the continuation of the job, low salary, a lack of power, protection, and rights (Kreshpaj et al., 2020; Rodgers & Rodgers, 1989; Standing, 2009; Utzet et al., 2020), vulnerability (Rodgers & Rodgers, 1989), lack of skill reproduction security (Standing, 2009) and restructuring and downsizing (Valero et al., 2021).

EP has repeatedly been shown to be associated with depression (Lopez et al., 2021), anxiety (Dawson et al., 2015; Lopez et al., 2021), suicide ideation, suicide attempts (Min et al., 2015) and various other adverse mental health outcomes (Jonsson et al., 2021). EP should be understood by considering the individual, household and national context in which EP and its consequences (Mai, 2017) occur and exist (Lain et al., 2019, Lain, Airey, Loretto, & Vickerstaff, 2020) as precarity in one of these three domains can exacerbate or weaken precarity in both other dimensions (Lain et al., 2019).

There is evidence suggesting that economic difficulty at an individual level is an explanatory mechanism for the association between EP and mental health (Ferrante G, Fasanelli F, Gigantesco A, Ferracin E, Contoli B, Costa G, Gargiulo L, Marra M, Masocco M, Minardi V, Violani C, Zengarini N, d’Errico A, Ricceri F., 2019; Fiori et al., 2016). Household poverty has been suggested as a potential moderator for the association between EP and mental health (Lain et al., 2019). However, studies which assess the potential moderation effect of poverty on household level are scarce.

The current evidence of a potential moderation effect of household poverty on the association between EP and mental health is largely based on qualitative research. For example, Lain et al. (Lain et al., 2019) found that participants in EP reported in interviews that precarious employment contributed to feelings of anxiety, especially amongst those who live in household poverty. Additionally, Clarke et al. (Clarke et al., 2007) found that not all people in jobs with high levels of EP have the same experience: participants who desired more secure jobs reported higher levels of anxiety and stress and more often lived in low-income households compared to their peers. In the only quantitative study on the moderating effect of household poverty, Han et al. (Han et al., 2017) found that household income level moderated the association between EP and suicide ideation amongst South-Korean employees. The study demonstrated that the association between EP and suicidal ideation is stronger in low and low-middle income households than in middle-high and high-income households (Han et al., 2017). The studies mentioned above have been conducted in a limited number of contexts: the United Kingdom (Lain et al., 2019), Canada (Clarke et al., 2007) and South Korea (Han et al., 2017). Thus, there is a need for quantitative studies which assess whether household poverty is a moderator for the association between EP and mental health in different contexts.

The welfare state is an important determinant for the association between EP and health (Kim et al., 2012), since it impacts labour market outcomes such as EP (Esping-Anderson, 1990; Jokela, 2017; MacMillan & Shanahan, 2021) and national policy and protection schemes negate or exacerbate the consequences of EP for health (Jokela, 2017). In Europe, the following welfare state types (WST) are commonly distinguished (Eikemo, Bambra, Judge, & Ringdal, 2008; Eikemo, Bambra, Joyce, & Dahl, 2008; Thévenon, 2011; Padrosa et al): Scandinavian, Bismarckian, Anglo-Saxon, South European and Central- and East European, based on welfare coverage (Ferrera, 1996)and development (Bonoli, 1997).Additionally, these welfare state types have different family policy (Thévenon, 2011) and gender norms related to the household (Eikemo & Bambra, 2008). We describe the similarities and differences between these WSTs in Box 1: European welfare state types. The heterogeneity between WSTs is mostly related to the extent to which welfare services and family support are provided. With regard to gender, Arias-de-la-Torr (Arias-de la Torre et al., 2019) argues that factors related to the financial support of the family are more important for men's mental health than women's, whereas Lain et al. (Lain et al., 2019) found that the financial situation of the household is of more importance to women as they usually have less resources of their own compared to men.

Box 1 **European welfare state types**

This categorization was based on Eikemo and Bambra (Eikemo & Bambra, 2008)who extended Fererra's typology (Ferrera, 1996) with an additional Central- and East European WST.

**South European type**.

In this WST, income maintenance is provided by a fragmented welfare system characterized by dualism: part of the workers in the regular ‘institutional’ labour market receive generous benefits whereas those working in the ‘non-institutional’ or ‘irregular’ market do not (Ferrera, 1996; Thévenon, 2011).This WST has a relatively high number of single-earner households (Thévenon, 2011)Traditional gender roles, in which the men are breadwinners (male-bread winner model) and the women take care of the children and the household (female care-giver model), are persistent (Moreno ).

**Central- and East European WST**.

The Central- and East European WST is characterized by relatively low benefits (Aidukaite, 2009; Bambra & Eikemo, 2009) and a history of Communist influence (Aidukaite, 2009; Eikemo & Bambra, 2008). The Central- and East European WST also has a relatively high number of single-earner households (Thévenon, 2011)and the male-breadwinner and female-caregiver models are persistent (Fodor et al., 2002; Pascall & Manning, 2000).

**Anglo-Saxon WST**.

In the Anglo-Saxon WST, welfare provision is limited and only provided when individuals meet strict criteria (Bambra & Eikemo, 2009). Mothers of young children often work part-time; thus, it is more common to find one and a half earners in the household compared to other WSTs (Thévenon, 2011).

**Bismarckian WST**.

Benefits in the Bismarckian type are commonly provided by the employer and do not aim to redistribute wealth (Eikemo & Bambra, 2008). The male bread-winner model in this WST has been replaced by a model similar to the Anglo-Saxon WST (Daiger Von et al., 2017).

**Scandinavian WST**.

The Scandinavian type is characterised by generous benefits and protection provided by the state (Bambra & Eikemo, 2009). Both men and women contribute to the household equally (Thévenon, 2011)Female- and maternal employment rates are highest in this WST (Thévenon, 2011)

Alt-text: Box 1

We expect that the moderating effect of household poverty on the association between EP and mental health may differ by gender and welfare state. The moderating effect of household poverty on the association between EP and mental health might be strongest in both South European and Central- and East European WSTs as welfare services are limited in both types, meaning that people living in household poverty may not be provided standard financial support by the state (Bambra & Eikemo, 2009).

In this study, we evaluate whether subjective household poverty moderates the association between employment precariousness (EP) and mental health across different types (WSTs): Scandinavian, South European, Central- and East European, Bismarckian and Anglo-Saxon and how this potential moderation effect differs for men and women within these WSTs. We hypothesize that the association between EP and mental health is stronger among individuals who experience household poverty compared to individuals who do not experience household poverty as living in household poverty might reinforce an individual's sense of experienced or subjective precarity (Lain et al., 2019) which is closely related to one's psychological well-being (Kiersztyn, 2017).

We use data from the sixth wave of the European Working Conditions Survey (EWCS), which was conducted in 2015. We estimate multi-level linear regression models with a random intercept, which we stratified by both gender and WST, to determine whether the association between EP and mental health is moderated by household poverty. To our knowledge, this is the first study to do so using data from Europe.

## 2 Data & methods

This study uses data from the sixth wave of the European Working Conditions Survey (EWCS-2015). The sample consists of 43,850 participants, across 35 European countries (Eurofound, 2017). We limited the analysis to 22 countries in which the Employment Precariousness Scale (EPRES-E), was found to be a reliable measure of employment precariousness (Padrosa et al., 2021): Austria, Belgium, Croatia, Denmark, Finland, France, Germany, Greece, Ireland, Italy, Lithuania, Luxembourg, the Netherlands, Norway, Poland, Portugal, Slovakia, Slovenia, Spain, Sweden, Switzerland and the United Kingdom (Padrosa et al., 2021). In the remaining 13 countries, the EPRES-E scale was shown to be unreliable (Padrosa et al., 2021). Thus, we have chosen to exclude participants from these countries. This left 29,621 participants in the sample. Participants who were aged 65 or over (N = 1245) were excluded from the sample, because the average normal retirement age in OECD countries in 2014 was 64.0 and 63.1 for men and women, respectively (OECD, 2015). Furthermore, the EPRES-E scale was developed for employees up until the age of 65 (Padrosa et al., 2021).

Additionally, participants who were self-employed, or refused to answer or answered ‘don't know’ when asked whether they were self-employed were excluded from the sample (N = 4357), as the EPRES-E scale was designed to include only formal employees and thus by definition excludes self-employment (Padrosa et al., 2021).

Participants who had no employment contract, participants who refused to answer or answered ‘other’ or ‘don't know’ when asked about their employment contract, were also excluded from the sample (N = 1271). This left 22,748 individuals in the sample.

Participants with missing data for any of the variables included in the EPRES-E scale (N = 3983) were excluded, because the individual EPRES-E score could not be calculated. The majority of the missing data for EP was due to missingness in the dimensions for wages (11,7%, N = 2678). Missing data for temporariness (1.44%), disempowerment (0.22%), vulnerability (2.88%), exercise of rights (1.87%) and unpredictability of working time (1.87%) were limited.

**Fig. 1:**
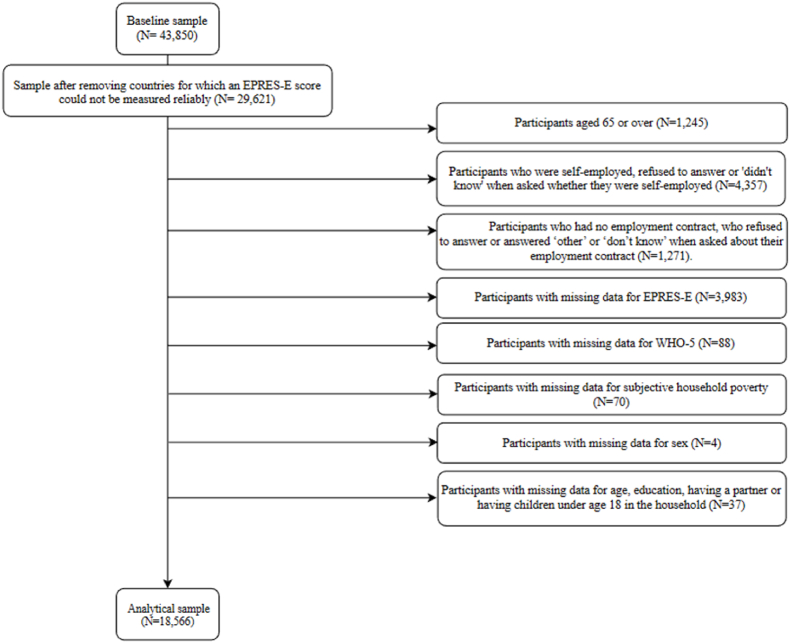
Flowchart of the in- and exclusion criteria, resulting in the analytical sample.

Additionally, we excluded participants with missing data on mental health (N = 88), subjective household poverty (N = 70) or sex (N = 4). Participants with missing data for age, education, having a partner or having children under age 18 in the household were also removed from the sample (N = 37). The analytical sample consisted of 18,566 participants, see Fig. 1.

### 2.1 Employment precariousness

EP was measured at the individual-level using the Employment Precariousness Scale Europe (EPRES-E). This scale consists of the following six dimensions: a) temporariness, b) disempowerment, c) vulnerability, d) wages, e) unpredictability of working hours and f) exercise of rights (Vives et al.). For each of the six dimensions mentioned above, a score ranging from 0 to 100 was created, weighted by the number of items within each dimension. The individual EPRES-E score was the mean score of the scores for each dimension, where a higher score refers to higher levels of EP.

### 2.2 Mental health

Mental health was measured using the World Health Organisation-Five Well-Being Index (WHO-5). The WHO-5 consists of five statements: over the past two weeks a) I have felt cheerful and in good spirits, b) I have felt calm and relaxed, c) I have felt active and vigorous, d) I woke up feeling fresh and rested and e) my life has been filled with things that interest me. The questions have six answer categories ranging from ‘all of the time’ to ‘at no time’, a score from 5 to 0, respectively, was assigned. All scores were added up and multiplied by 4 to create a continuous scale ranging from 0 to 100, where a higher score refers to better mental health.

### 2.3 Subjective household poverty

Subjective household poverty was measured by a single item: ‘Is your household able to make ends meet?’ with the following answer categories: a) very easily, b) easily, c) fairly easily, d) with some difficulty, e) with difficulty and f) with great difficulty. This variable was dichotomized: participants who answered ‘with great difficulty’ or ‘with difficulty’ were considered to be living in household poverty (Filandri et al., 2020; Mai et al., 2019).

### 2.4 Welfare states typology

The included countries were categorized into five WST groups: a) Scandinavian: Denmark, Finland, Norway and Sweden, b) South European: Greece, Italy, Portugal and Spain c) Central- and East European: Croatia, Lithuania, Poland, Slovakia and Slovenia d) Bismarckian: Austria, Belgium, France, Germany, Netherlands, Luxembourg, Switzerland, and e) Anglo-Saxon: Ireland and The United Kingdom (Eikemo & Bambra, 2008; Ferrera, 1996).

### 2.5 Covariates

Based on previous studies, we included the following covariates: having a partner (Ferrante et al., 2019)having children (Ferrante et al., 2019; Fiori et al., 2016)age and education (Ferrante et al., 2019; Fiori et al., 2016; Mai, 2017; Méndez Rivero et al., 2021). We created a binary variable for having a partner (yes/no) and for having children under age 18 in the household (yes/no). A categorical variable for education was created based on the International Standard Classification of Education: primary (early childhood education and primary education), secondary (lower or upper secondary education or post-secondary non-tertiary education) or tertiary (short-cycle tertiary education, or a Bachelor's, Master's or doctorate degree).

### 2.6 Power calculation

We performed power calculations in R-Studio (version 4.40), using the simr package after estimating the models with lme4. Since using expected effect sizes for all model parameters would be overly complex and incomprehensible because of the high number of parameter (Lane & Hennes, 2018), we only used an expected effect size (of-0.5) for the interaction term. For the other parameters we used observed effect sizes. To obtain a more precise estimate of the power to detect this interaction effect, we conducted 1000 simulations. The power of our different models, using this expected effect-size of the interaction term, can be found in Appendix II and ranged from 57.50% to 100% for men and from 56,50%–100% for women (depending on the type of welfare state).

### 2.7 Statistical analysis

All models were stratified by WST and gender. We estimated two types of multilevel models to assess the association between EP and mental health, moderated by household poverty. First, multi-level models with an interaction term between household poverty and EP and a random intercept for EP at country level (M1) were estimated. Second, multilevel models with an interaction term between household poverty and EP with both a random intercept for country and random slope for EP (M2) were estimated to determine whether the association between EP and mental health scores differed across countries within a WST. Likelihood-ratio tests (LRT) were performed to determine which models (M1 or M2) fit the data best, i.e. whether the addition of a random slope improved model fit. If the LRT had a p-value lower than 0.05, this meant that the addition of a random slope did not significantly improve the model.To ensure comparability, we chose to use the same kind of model (M1 or M2) across all WSTs and both genders. The addition of a random slope did not significantly improve most models. Thus, the intercept-only models (M1) were chosen and the slope was fixed across all countries within a WST.

To assess the robustness of the results, subjective household poverty was dichotomized as experiencing no difficulty versus experiencing difficulty in a sensitivity analysis.

In some of the WSTs, small subsamples exist in certain categories of the covariates, which may limit the power of our analyses. As a robustness check, we also estimated models for a combined sample of welfare state types, both for a combined sample of men and women and stratified by gender. We estimated multi-level models with an interaction term between household poverty and EP and a random intercept for EP at the level of the welfare state type (M1). We also estimated multilevel models with an interaction term between household poverty and EP with both a random intercept for welfare state type and random slope for EP (M2). Likelihood-ratio tests (LRT) were performed to determine which models fit the data for the full combined sample best. We repeated these analysis for the combined sample of welfare state types, but stratified by gender. For the full, combined sample of men and women, and the sex-specific models, the addition of a random slope significantly improved the models (M2). Thus, we have chosen models with both a random intercept and random slope at the level of the welfare state type. All analyses were performed in STATA (version 17.0).

## 3 Results

### 3.1 Descriptive characteristics

The descriptive statistics of the sample can be found in Table 1. Average mental health scores were highest in the South European (73.36 and 71.03 for men and women, respectively), followed by Scandinavian (71.58 and 68.37), Bismarckian (70.23 and 67.20), Central- and East European (68.42 and 66.82) and Anglo-Saxon (66.35 and 65.24) WST. Mental health scores were slightly better amongst men than women in all WSTs.

**Table 1:** Sociodemographic characteristics of the sample, overall and stratified by welfare state type.

	Total		Scandinavian		South European		Central- and East European		Bismarckian		Anglo-Saxon	
	Men (N = 8778)	Women (N = 9788)	Men (N = 1485)	Women (N = 1697)	Men (N = 1584)	Women (N = 1625)	Men (N = 1476)	Women (N = 1987)	Men (N = 3452)	Women (N = 3672)	Men (N = 781)	Women (N = 807)
WHO-5												
*Mean* (SD)	70.37 (19.27)	67.80 (20.37)	71.58 (16.38)	68.37 (17.97)	73.36 (19.08)	71.03 (19.77)	68.42 (19.68)	66.82 (20.55)	70.23 (19.71)	67.20 (21.05)	66.35 (20.79)	65.24 (21.97)
EPRES-E												
*Mean* (SD)	29.41 (12.52)	30.88 (12.21)	25.87 (11.09)	28.53 (11.03)	31.04 (13.30)	31.68 (12.97)	32.96 (12.68)	32.75 (12.26)	28.75 (12.16)	30.75 (12.04)	28.98 (12.48)	30.24 (12.70)
Household poverty												
*No*	7950 (90.57%)	8678 (88.66%)	1447 (97.44%)	1657 (97.64%)	1273 (80.37%)	1307 (80.43%)	1308 (88.62%)	1675 (84.30%)	3190 (92.41%)	3300 (89.87%)	732 (93.73%)	739 (91.57%)
*Yes*	828 (9.43%)	1110 (11.34%)	38 (2.56%)	40 (2.36%)	311 (19.63%)	318 (19.57%)	168 (11.38%)	312 (15.70%)	262 (7.59%)	372 (10.13%)	49 (6.27%)	68 (8.43%)
Age												
*Mean* (SD)	42.19 (11.54)	42.41 (11.31)	43.25 (11.90)	43.61 (12.37)	41.96 (10.92)	42.01 (10.61)	41.71 (11.64)	42.97 (10.86)	42.07 (11.58)	41.97 (11.25)	42.03 (11.64)	41.32 (11.41)
Education												
*Primary*	292 (3.33%)	261 (2.67%)	9 (0.61%)	19 (1.12%)	171 (10.80%)	136 (8.37%)	2 (0.14%)	2 (0.10%)	94 (2.72%)	93 (2.53%)	16 (2.05%)	11 (1.36%)
*Secondary*	5734 (65.32%)	5636 (57.58%)	837 (56.36%)	716 (42.19%)	1032 (65.15%)	983 (60.49%)	1119 (75.81%)	1239 (62.36%)	2340 (67.79%)	2317 (63.10%)	406 (51.98%)	381 (47.21%)
*University*	2752 (31.35%)	3891 (39.75%)	639 (43.03%)	962 (56.69%)	381 (24.05%)	506 (31.14%)	355 (24.05%)	745 (37.54%)	1018 (29.49%)	1262 (34.37%)	359 (45.97%)	415 (51.43%)
Children <18 in the household												
*No*	7766 (88.47%)	8235 (84.13%)	1326 (89.29%)	1519 (89.51%)	1372 (86.62%)	1324 (81.48%)	1253 (84.89%)	1522 (76.60%)	3104 (89.92%)	3169 (86.30%)	711 (91.04%)	701 (86.86%)
*Yes*	1012 (11.53%)	1553 (15.87%)	159 (10.71%)	178 (10.49%)	212 (13.38%)	301 (18.52%)	223 (15.11%)	465 (23.40%)	348 (10.08%)	503 (13.70%)	70 (8.96%)	106 (13.14%)
Partner												
*No*	2926 (33.33%)	3636 (37.15%)	436 (29.36%)	572 (33.71%)	574 (36.24%)	639 (39.32%)	484 (32.79%)	649 (32.66%)	1191 (34.50%)	1450 (39.49%)	241 (30.86%)	326 (40.40%)
*Yes*	5852 (66.67%)	6152 (62.85%)	1049 (70.64%)	1125 (66.29%)	1010 (63.76%)	986 (60.68%)	992 (67.21%)	1338 (67.34%)	2261 (65.50%)	2222 (60.51%)	540 (69.14%)	481 (59.60%)

*SD* Standard deviation.

*N* Number of participants.

Household poverty was most common in the South European (19.63% and 19.57% for men and women), followed by Central- and East European (11.38% and 15.70%), Bismarckian (7.59% and 10.13%), Anglo-Saxon (6.27% and 8.43%) and Scandinavian (2.56% and 2.36%) WST. In all WSTs, women lived in household poverty more often.

EP scores were highest in Central- and East European WST (32.96 and 32.75 for men and women, respectively), followed by the South European WST countries (31.04 and 31.68). The Anglo-Saxon and Bismarckian WSTs had similar scores: for men (28.75 versus 28.98, respectively) and women (30.75 versus 30.24, respectively). Scandinavian men and women had the lowest mean EP scores (25.87 and 28.53). Women's EP scores were higher in all WSTs, except the Central- and East European WST.

While there are differences between the characteristics of the included participants and the excluded participants, no consistent differences between the samples can be observed across the welfare state types, see Appendix I.

#### 3.1.1 Final models

We present fully adjusted models with a random intercept at country-level, which were adjusted for age, education, having a partner and having children under age 18. Additionally, we created interaction plots for these models. These interaction plots visualize the linear association between EP and mental health, for individuals who do and do not live in subjective household poverty. All covariates were set to their means, thus, the random intercept was not shown.

**Fig. 2:**
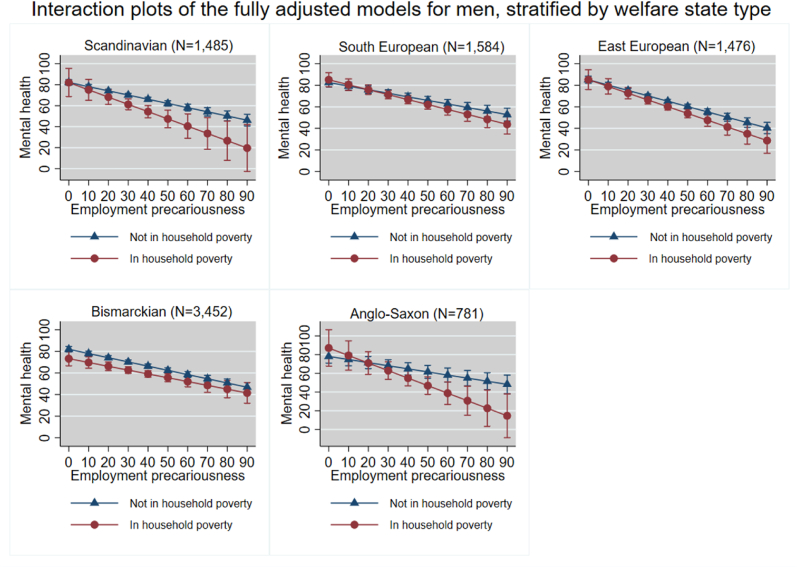
Interaction plots of the association between EP and mental health amongst men, stratified by subjective household poverty for each welfare state type, separately.

Table 2 shows that there was a negative association between EP and mental health in all WSTs. There was no moderating effect of household poverty on the association between EP and mental health in the different WSTs, except for the Anglo-Saxon WST where household poverty was a statistically significant negative moderator (−0.47, 95% CI -0.93; −0.02). This is also visualized by the interaction graphs, see Fig. 2. Fig. 2 demonstrates that differences in mental health scores between men in household poverty and men not in household poverty are largest in the Scandinavian and Anglo-Saxon WST. Minimal differences exist in the South European and Central- and East European WSTs.

**Table 2:** Final models for the association between EP and mental health amongst men moderated by subjective household poverty, stratified by welfare state type. Adjusted for age, education, having a partner and having children under age 18.

Men (N = 8778)	Scandinavian (N = 1485)	South European (N = 1584)	Central- and East European (N = 1476)	Bismarckian (N = 3452)	Anglo-Saxon (N = 781)
Fixed effects					
Intercept (95% CI)	85.82 (74.23; 97.41)	100.62 (93.26; 107.98)	103.40 (77.05; 129.74)	89.22 (83.45; 94.99)	90.65 (76.31; 104.98)
Employment precariousness (95% CI)	−0.40 (−0.48; −0.32)	−0.33 (−0.41; −0.25)	−0.50 (−0.58; −0.41)	−0.39 (−0.45; −0.33)	−0.33 (−0.46; −0.21)
Household poverty (95% CI)	−0.09 (−13.55; 13.37)	2.59 (−3.75; 8.94)	0.08 (−9.39; 9.54)	−8.71 (−15.45; −1.98)	8.96 (−9.86; 27.78)
Household poverty∗Employment precariousness (95% CI)	−0.29 (−0.68; 0.09)	−0.13 (−0.30; 0.04)	−0.13 (−0.37; 0.11)	0.04 (−0.14; 0.21)	−0.47 (−0.93; −0.02)
Random effects					
Random intercept (Standard error)	1.75 (1.70)	10.06 (7.81)	2.21 (2.23)	5.17 (3.28)	19.99 (21.13)
Residual variance (Standard error)	243.11 (8.93)	326.59 (11.62)	338.10 (12.47)	360.14 (8.68)	383.43 (19.43)
Intraclass correlation coefficient (95% CI)∗	0.00 (0.00; 0.04)	0.03 (0.01; 0.13)	0.01 (0.00; 0.05)	0.01 (0.00; 0.04)	0.04 (0.01; 0.27)

*N* Number of participants.

∗ ICC of constant-only model.

**Fig. 3:**
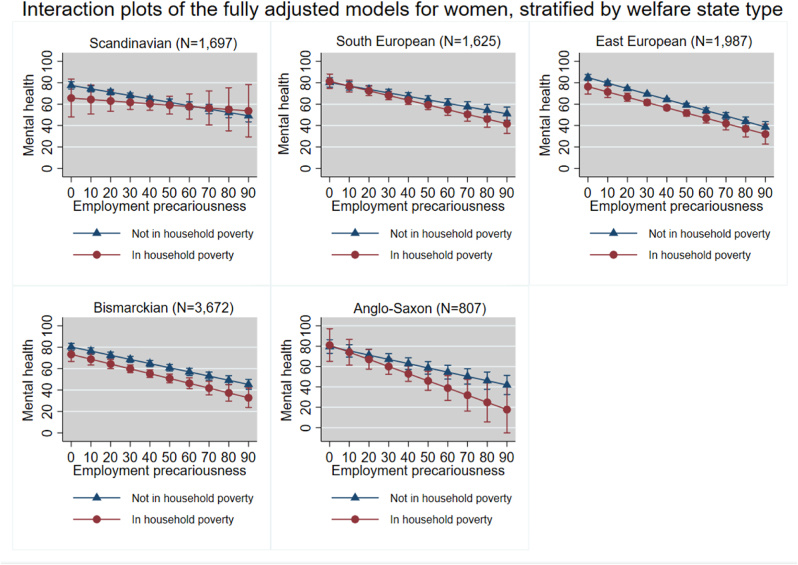
Interaction plots of the association between EP and mental health amongst women, stratified by subjective household poverty for each welfare state type, separately.

Table 3 shows that a negative association between EP and mental health was found in all WSTs. No moderating effect of household poverty on the association between EP and mental health was found in any of the WSTs. The plots in Fig. 3 suggest that the negative effect of living in household poverty on the association between EP and mental health scores in these WSTs is biggest in the Anglo-Saxon WST, although this effect is minimal.

**Table 3:** Final models for the association between EP and mental health amongst women moderated by subjective household poverty, stratified by welfare state type. Adjusted for age, education, having a partner and having children under age 18.

Women (N = 9788)	Scandinavian (N = 1697)	South European (N = 1625)	Central- and East European (N = 1987)	Bismarckian (N = 3672)	Anglo-Saxon (N = 807)
Fixed effects					
Intercept (95% CI)	80.22 (70.43; 90.02)	89.80 (81.75; 97.85)	94.97 (67.86; 122.07)	87.38 (81.06; 93.70)	90.46 (74.48; 106.44)
Employment precariousness (95% CI)	−0.32 (−0.40; −0.23)	−0.32 (−0.41; −0.24)	−0.51 (−0.60; −0.43)	−0.39 (−0.45; −0.33)	−0.42 (−0.54; −0.29)
Household poverty (95% CI)	−11.84 (−29.56; 5.89)	1.03 (−5.42; 7.48)	−8.47 (−15.87; −1.07)	−6.98 (−13.33; −0.63)	1.49 (−14.10; 17.07)
Household poverty∗Employment precariousness (95% CI)	0.18 (−0.27; 0.64)	−0.12 (−0.29; 0.05)	0.02 (−0.17; 0.21)	−0.06 (−0.23; 0.11)	−0.28 (−0.70; 0.13)
Random effects					
Random intercept (Standard error)	5.20 (4.19)	10.48 (8.11)	0.00 (0.00; 0.00)	13.68 (7.85)	15.06 (16.25)
Residual variance (Standard error)	303.70 (10.44)	352.75 (12.39)	369.11 (11.71)	395.37 (9.24)	425.56 (21.21)
Intraclass correlation coefficient (95% CI)∗	0.01 (0.00; 0.07)	0.03 (0.01; 0.12)	0.00 (0.00; 0.04)	0.04 (0.01; 0.10)	0.04 (0.00; 0.23)

*N* Number of participants.

∗ ICC of constant-only model.

#### 3.1.2 Sensitivity analysis

For both men and women, the differences in mental health scores between participants living in household poverty and participants not living in household poverty were smaller in the robustness check, see Appendix II.

In the full sample, not stratified by welfare state type, we found a statistically significant moderation effect of household poverty on the association between EP and mental health for the combined sample of men and women (−0.08, 95% CI -0.15; −0.01) see Appendix IV. However, the difference is small and may not be clinically relevant.

We did not, however, find a statistically significant moderation effect of household poverty on the association between EP and mental health in the sex-specific models for men (−0.09, 95% CI -0.19; 0.01) or for women (−0.08, 95% CI -0.17; 0.02) see Appendix IV. The moderation effect of subjective household poverty on the association between EP and mental health is thus slightly stronger for men, which is also reflected in the interaction plots, see Appendix V.

To visualize how the relationship between EP and mental health amongst people living in household poverty and people not living in household poverty differs across welfare state types, we created plots for both the full, combined sample of men and women and the sex-specific models of the full sample, see Appendix VI.

## 4 Discussion

In this study, we investigated whether subjective household poverty moderates the association between EP and mental health. We expected that the association between EP and mental health would be stronger amongst people living in household poverty compared to people not living in household poverty, and that this effect would be strongest in the South European and East European WSTs.

We consistently found that EP is negatively related to mental health, which is in line with previous research (Rönnblad et al., 2019; Utzet et al., 2020). Regarding household poverty, the main findings of this study indicate that the association between EP and mental is only slightly more negative for those who experience household poverty. We only found a statistically significant moderation effect of subjective household poverty on the association between EP and mental health amongst men in the Anglo-Saxon WSTs. In the full, combined sample of both men and women we did find a statistically significant moderation effect of subjective household poverty on the association between EP and mental health. However, this effect is small and it is doubtful whether it can be considered clinically relevant.

The results of the analysis conducted on the combined sample of men and women, not stratified by WSTs, show a small but statistically significant moderation effect, which is consistent with previous research. For example, the study performed by Han et al. (Han et al., 2017), demonstrated that household income mediated the association between EP and suicide ideation in Korea. Furthermore, evidence from qualitative studies suggested that subjective household poverty moderates the association between EP and mental health. For example, Clarke (Clarke et al., 2007) found that participants in precarious employment who desired more stable jobs had higher levels of stress and anxiety and more often lived in low-income households. Additionally, Lain et al. (Lain et al., 2019) found that household poverty can act as a buffer against or catalyst for precarity, especially for women. The effect size found in our study, however, is small and thus, the negative moderation effect of household poverty on the association between EP and mental health may not be clinically relevant. The significant moderation effect found in the full sample, might be attributed to increased statistical power due to the large sample size rather than the fact that a moderation effect between EP and mental health exists in all WSTs. This is further substantiated by the fact that we were able to detect a statistically moderation effect of subjective household poverty on the association between EP and mental health amongst men in the Anglo-Saxon WST, which was the smallest sub-sample in this study, suggesting that a moderation effect truly exists in this sub-sample.

A potential explanation for the absence of a statistically significant interaction effect of household poverty in all of the WSTs apart from the Anglo-Saxon WST may be found in differences in welfare state policies and the role of family. In the Bismarckian and Scandinavian WSTs, protection schemes are relatively high. Although welfare services are limited in the South- and Central- and East European WSTs, one can rely on family members for financial support when unable to make ends meet (Eikemo, Bambra, Judge, & Ringdal, 2008; Aidukaite, 2009; den Dulk et al., 2012). South European countries have a family-focused culture in which the extended family has often fulfilled the need for welfare services by providing a basic income to its members (den Dulk et al., 2012; Moreno et al., 2013; Ferrera and Ferrera, 2005). Less is known about the Central- and East European WST, but during the post-Communist transition the family was still seen as an institution providing resources (Vives et al.). The negative moderating effect might be felt less by people living in household poverty in the Bismarckian, Scandinavian, South- and Central- and East European WST because of the external help from welfare services and family members they receive, whereas they might still report living in subjective household poverty (Filandri et al., 2020). This is in contrast to the Anglo-Saxon welfare state type where welfare services are limited (Bambra & Eikemo, 2009) and the role of family is not emphasized (Eikemo & Bambra, 2008).

Our study has several strengths. This study used a theoretical framework formulated by Lain et al. (Lain et al., 2019) in which factors on individual, household and national level interact and shape the context in which EP exists. By using the framework, we aimed to expand understanding of the association between EP and mental health outside of the individual employment relationship. Thus, we used a European welfare state typology as formulated by Eikemo and Bambra (Eikemo & Bambra, 2008) to consider how these welfare state types might affect a potential moderation effect of subjective household poverty on the association between EP and mental health. Additionally, we stratified for gender as gender norms and family-policy differ across these WSTs (Eikemo & Bambra, 2008; Thévenon, 2011).

Second, the national context plays an important role for both EP and its mental health consequences (Lain et al., 2019, 2020; Mai, 2017). In this study, we use EPRES-E which was formulated to enable cross-country comparison of EP across 22 European countries (Padrosa et al., 2021). This allowed us to investigate a potential moderating role of subjective household poverty on the association between EP and mental health in a broader context, thereby providing more insight into this association.

Lastly, this study used a subjective definition of household poverty based on whether the household is able to make ends meet. Often, either objective or relative measures of (household) poverty are used, in which people below a certain threshold are considered to be living in poverty (Bradshaw & Nieuwenhuis, 2021). We have chosen to define household poverty based on subjective experience as we expect that this is more sensitive to individual experiences compared to objective and relative measure of household poverty (Filandri et al., 2020).

Our study is subject to several limitations and thus, the results need to be interpreted with caution. Given the cross-sectional nature of the study, no conclusions can be drawn regarding the direction of the association between employment precariousness and mental health. The association between EP and mental health is bidirectional as people in employment are healthier than the general population as people with better health are more often hired and more likely to stay in the workforce (Wagenaar et al., 2012). Thus, no conclusions can be drawn regarding the direction of the association between employment precariousness and mental health.

Second, the sample contained 17.5% (N = 3983) respondents with missing data for EP due to missingness in one or several of the six dimensions of EP. While there are differences between the descriptive characteristics of the included participants and the excluded participants, see Appendix II, no consistent differences between the samples can be observed across the welfare state types. This suggests that the exclusion of the participants with missing data for any of the items of the EPRES-E scale, did not affect the results of our study.

Third, we needed to exclude a number of countries (Albania, Bulgaria, Cyprus, Czech Republic, Estonia, Hungary, Latvia, Malta, Montenegro, Republic of North Macedonia, Romania, Serbia and Turkey) from our analyses, because the EPRES-E was proven to be unreliable in these countries. This could, have led to overestimation of the association between EP and mental health and the role of household poverty in the analyses conducted on the full, combined sample. Our analyses suggest that, amongst women in the Central- and East European welfare state type subjective household poverty does not have a non-statistically significant negative effect on the association between EP and mental health (0.02, 95% CI -0.17; 0.21). Rather, the results seem to suggest the opposite, although the differences are negligible. This could mean that inclusion of the excluded countries, particularly Estonia, Latvia, Hungary Bulgaria, Czech Republic and Romania, which are sometimes considered to be included in the Central- and Eastern welfare state type (Fenger, 2007; Lauzadyte-Tutliene et al., 2018) would lead to a diminished moderation effect of household poverty on the association between EP and mental health.

Fourthly, since the effect size of the interaction term was smaller than we hypothesized, our sample size was not sufficient to detect the observed differences in the association between employment precariousness and mental health amongst those living in household poverty and those not living in household poverty. However, the power of our analyses was sufficient to detect the expected effect size in the majority of welfare state types.

Finally, EPRES-E is limited to waged and salaried workers, which means that people in informal work, who arguably have the jobs with the highest levels of employment precariousness, have been excluded from the sample (Padrosa et al., 2021). In both the South European and the Central- and East European WSTs these forms of informal employment are more common than in the other WSTs (Padrosa et al., 2021).

Our study was performed with data from the sixth wave of the European Working Conditions Survey (EWCS-2015). The sample used in the EWCS-2015 is representative of the working population aged 15 and over (16 and over in Bulgaria, Norway, Spain and the United Kingdom) residing in the 35 countries included in the survey (Ipsos) . It is thus expected that the results of our study can be generalized to the working population in the included countries. However, it is unclear whether the results of our study are generalizable to a broader context. The EPRES-E is reliable in Western, post-industrialist countries and should only be used in such contexts as the scale thus might not capture employment precariousness in different socio-political environments (Padrosa et al., 2021). Thus, future research should investigate whether subjective household poverty moderates the association between EP and mental health in different institutional contexts, especially since previous research from Korea (Han et al., 2017) suggest that such a moderation effect might exist.

## 5 Conclusion

In summary, this study assessed whether household poverty is a moderator in the association between employment precariousness and mental health. A moderating effect of household poverty on the association between employment precariousness and mental health was demonstrated amongst men in the Anglo-Saxon welfare state type and a combined full sample of men and women. Further research is advised to ensure better understanding of the mechanisms underlying the association between employment precariousness and mental health.

## Ethical statement

Hereby we declare that the manuscript is our own original work, which has not been previously published elsewhere.

We declare that the manuscript is not currently being considered for publication elsewhere.

The manuscript reflects our own research and analysis in a truthful and complete manner. The manuscript properly credits the meaningful contributions of co-authors and co-researchers.

The results are appropriately placed in the context of prior and existing research.

All sources used are properly disclosed.

All authors have been personally and actively involved in substantial work leading to the paper, and will take public responsibility for its content.

Ethical approval was not applicable in this study because we utilized secondary data and did not collect or record new information. Data from the European Working Conditions Survey 2015 is available from UK Data Service: http://doi.org/10.5255/UKDA-Series-200014.

## CRediT authorship contribution statement

**Ceciel Pauls:** Writing – original draft, Methodology, Formal analysis, Conceptualization. **Maria Fleischmann:** Writing – review & editing, Supervision, Methodology, Conceptualization. **Michel Klein:** Writing – review & editing, Supervision, Conceptualization. **Stef Bouwhuis:** Writing – review & editing, Supervision, Methodology, Conceptualization. **Judith E. Bosmans:** Writing – review & editing, Supervision, Methodology, Conceptualization.

## Declaration of competing interest

The authors declare that they have no known competing financial interests or personal relationships that could have appeared to influence the work reported in this paper.

## Figures and Tables

**Table 4:** Comparison of the sociodemographic characteristics of the excluded and included sample of men, stratified by welfare state type.

	Complete	Incomplete	P-value	Complete	Incomplete	P-value	Complete	Incomplete	P-value	Complete	Incomplete	P-value	Complete	Incomplete	P-value
	N = 1485	N = 105		N = 1584	N = 602		N = 1476	N = 497		N = 3452	N = 629		N = 781	N = 175	
WHO-5^1^															
*Mean* (SD)	71.58 (16.38)	71.37 (19.50)	0.92	73.36 (19.08)	71.13 (20.10)	0.02	68.42 (19.68)	67.72 (19.51)	0.50	70.23 (19.71)	71.87 (18.91)	0.05	66.35 (20.79)	71.76 (21.47)	0.00
EPRES-E^1^															
*Mean* (SD)	25.87 (11.09)	36.67 (14.82)	0.03	31.04 (13.30)	38.39 (14.63)	0.11	32.96 (12.68)	38.62 (12.96)	0.05	28.75 (12.16)	30.33 (12.24)	0.52	28.98 (12.48)	35.27 (15.93)	0.15
Household poverty^2^															
*No*	97.44%	97.87%		80.37%	82.40%		88.62%	87.76%		92.41%	92.41%		93.73%	93.98%	
*Yes*	2.56%	2.13%	0.80	19.63%	17.60%	0.29	11.38%	12.24%	0.61	7.59%	7.59%	1.00	6.27%	6.02%	0.90
Age^1^															
*Mean* (SD)	43.25 (11.90)	41.81 (14.88)	0.33	41.96 (10.92)	43.30 (10.60)	0.01	41.71 (11.64)	42.78 (11.45)	0.07	42.07 (11.58)	42.91 (12.10)	0.11	42.03 (11.64)	42.10 (12.59)	0.95
Education^2^															
*Primary*	0.61%	0.00%		10.80%	5.71%		0.14%	0.41%		2.72%	3.24%		2.05%	1.28%	
*Secondary*	56.36%	68.63%		65.15%	63.19%		75.81%	69.59%		67.79%	66.45%		51.98%	54.49%	
*University*	43.03%	31.37%	0.05	24.05%	31.09%	0.00	24.05%	30.00%	0.02	29.49%	30.31%	0.68	45.97%	44.23%	0.73
Children younger than 18 in the household^2^															
*No*	89.29%	94.29%		86.62%	85.22%		84.89%	82.09%		89.92%	87.60%		91.04%	89.14%	
*Yes*	10.71%	5.71%	0.11	13.38%	14.78%	0.40	15.11%	17.91%	0.14	10.08%	12.40%	0.08	8.96%	10.86%	0.44
Partner^2^															
*No*	29.36%	44.76%		36.24%	30.90%		32.79%	35.21%		34.50%	33.86%		30.86%	30.29	
*Yes*	70.64%	55.24%	0.00	63.76%	69.10%	0.02	67.21%	64.79%	0.32	65.50%	66.14%	0.76	69.14%	69.71%	0.88

*SD* Standard deviation.

*N* Number of participants.

Note: Tests were performed with the available data from the participants with incomplete data.

1 Assessed with *t*-test for unequal variances.

2 Assessed with chi-squared test.

**Table 5:** Comparison of the sociodemographic characteristics of the excluded and included sample of women, stratified by welfare state type.

	Complete	Incomplete	P-value	Complete	Incomplete	P-value	Complete	Incomplete	P-value	Complete	Incomplete	P-value	Complete	Incomplete	P-value
	N = 1697	N = 106		N = 1625	N = 631		N = 1987	N = 575		N = 3672	N = 696		N = 807	N = 162	
WHO-5^1^															
*Mean* (SD)	68.37 (17.97)	66.87 (18.77)	0.43	71.03 (19.77)	68.71 (21.05)	0.02	66.82 (20.55)	64.57 (20.18)	0.02	67.20 (21.05)	70.15 (20.43)	0.00	65.24 (21.97)	67.38 (21.87)	0.26
EPRES-E^1^															
*Mean* (SD)	28.53 (11.03)	42.53 (13.44)	0.01	31.68 (12.97)	35.08 (12.07)	0.18	32.75 (12.26)	33.71 (12.28)	0.65	30.75 (12.04)	37.55 (14.85)	0.01	30.24 (12.70)	30.83 (15.38)	0.93
Household poverty^2^															
*No*	97.64%	94.00%		80.43%	84.69%		84.30%	83.91%		89.87%	89.29%		91.57%	91.56%	
*Yes*	2.36%	6.00%	0.03	19.57%	15.31%	0.02	15.70%	16.09%	0.83	10.13%	10.71%	0.65	8.43%	8.44%	1.00
Age^1^															
*Mean* (SD)	43.61 (12.37)	42.01 (14.25)	0.26	42.01 (10.61)	42.46 (10.23)	0.36	42.97 (10.86)	43.00 (10.64)	0.95	41.97 (11.25)	42.06 (11.32)	0.86	41.32 (11.41)	44.02 (11.22)	0.10
Education^2^															
*Primary*	1.12%	0.00%		8.37%	6.08%		0.10%	0.18%		2.53%	5.26%		1.36%	1.27%	
*Secondary*	42.19%	57.28%		60.49%	55.84%		62.36%	64.56%		63.10%	63.01%		47.21%	57.59%	
*University*	56.69%	42.72%	0.01	31.14%	38.08%	0.00	37.54%	35.26%	0.56	34.37%	31.73%	0.00	51.43%	41.14%	0.06
Children younger than 18 in the household^2^															
*No*	89.51%	87.74%		81.48%	80.82%		76.60%	78.26%		86.30%	87.93%		86.86%	89.51%	
*Yes*	10.49%	12.26%	0.57	18.52%	19.18%	0.72	23.40%	21.74%	0.40	13.70%	12.07%	0.25	13.14%	10.49%	0.36
Partner^2^															
*No*	33.71%	40.57%		39.32%	35.50%		32.66%	34.43%		39.49%	40.23%		40.40%	40.74%	
*Yes*	66.29%	59.43%	0.15	60.68%	64.50%	0.09	67.34%	65.57%	0.43	60.51%	59.77%	0.71	59.60%	59.26%	0.94

Note: Tests were performed with the available data from the participants with incomplete data.

*SD* Standard deviation.

*N* Number of participants.

1 Assessed with *t*-test for unequal variances.

2 Assessed with chi-squared test.

**Table 6:** Power of the expected interaction effect (−0.5), given our sample of men.

Men	Scandinavian (N = 1485)	South European (N = 1584)	Central- and East European (N = 1476)	Bismarckian (N = 3452)	Anglo-Saxon (N = 781)
Power of the expected effect size (95% CI)	71.10% (68.18; 73.89)	99.90% (99.44; 100.00)	98.30% (97.29; 99.01)	100.0% (99.63; 100.0)	57.50% (54.37; 60.59)

**Table 7:** Power of the expected interaction effect (−0.5), given our sample of women.

Women	Scandinavian (N = 1697)	South European (N = 1625)	Central- and East European (N = 1987)	Bismarckian (N = 3672)	Anglo-Saxon (N = 807)
Power of the expected effect size (95% CI)	56.50% (53.36; 59.60)	100.0% (99.63; 100.0)	99.90% (99.44; 100.00)	100.0% (99.63; 100.0)	66.50% (63.48; 69.42)

**Table 8:** Power of the expected interaction effect (−0.5), given the sample of men, women and the combined full sample.

	Full sample (N = 18,566)	Men (N = 8778)	Women (N = 9788)
Power of the expected effect size (95% CI)	100.0% (99.63, 100.0)	100.0% (99.63, 100.0)	100.0% (99.63, 100.0)

**Table 9:** Models for the association between EP and mental health amongst men moderated by subjective household poverty, stratified by welfare state type. Adjusted for age, education, having a partner and having children under age 18.

Men (N = 8778) Fully adjusted	Scandinavian (N = 1485)	South European (N = 1584)	Central- and East European (N = 1476)	Bismarckian (N = 3452)	Anglo-Saxon (N = 781)
Fixed effects					
Intercept (95% CI)	86.17 (74.58; 97.75)	101.36 (93.70; 109.03)	101.81 (75.49; 128.14)	90.46 (84.64; 96.28)	90.55 (76.07; 105.03)
Employment precariousness (95% CI)	−0.40 (−0.49; −0.31)	−0.32 (−0.43; −0.21)	−0.41 (−0.52; −0.30)	−0.36 (−0.43; −0.28)	−0.32 (−0.46; −0.18)
Household poverty (95% CI)	−7.85 (−14.85; −0.86)	−0.77 (−5.47; 3.93)	3.75 (−1.85; 9.35)	−6.57 (−10.51; −2.64)	3.95 (−5.15; 13.05)
Household poverty∗Employment precariousness (95% CI)	0.04 (−0.17; 0.25)	−0.05 (−0.19; 0.09)	−0.21 (−0.37; −0.06)	−0.00 (−0.12; 0.11)	−0.24 (−0.50; 0.02)
Random effects					
Random intercept (Standard error)	1.54 (1.55)	10.12 (7.87)	2.08 (2.14)	4.67 (3.02)	20.23 (21.41)
Residual variance (Standard error)	241.82 (8.89)	326.32 (11.61)	336.44 (12.41)	355.91 (8.58)	386.74 (19.60)
Intraclass correlation coefficient (95% CI)∗∗	0.00 (0.00; 0.04)	0.03 (0.01; 0.13)	0.01 (0.00; 0.05)	0.01 (0.00; 0.04)	0.04 (0.01; 0.27)

*N* Number of participants.

∗ Household poverty was recoded: those who experience no difficulty versus those who experience difficulty.

∗∗ ICC of constant-only model.

**Table 10:** Final models for the association between EP and mental health amongst women moderated by subjective household poverty, stratified by welfare state type. Adjusted for age, education, having a partner and having children under age 18.

Women (N = 9788) Fully adjusted	Scandinavian (N = 1697)	South European (N = 1625)	Central- and East European (N = 1987)	Bismarckian (N = 3672)	Anglo-Saxon (N = 807)
Fixed effects					
Intercept (95% CI)	81.11 (71.25; 90.97)	92.30 (83.81; 100.78)	94.89 (67.78; 122.01)	88.28 (81.89; 94.66)	90.91 (74.61; 107.20)
Employment precariousness (95% CI)	−0.31 (−0.40; −0.22)	−0.36 (−0.47; −0.24)	−0.51 (−0.62; −0.40)	−0.33 (−0.41; −0.26)	−0.43 (−0.58; −0.29)
Household poverty (95% CI)	−5.76 (−13.23; 1.71)	−3.42 (−8.42; 1.58)	−6.95 (−11.96; −1.95)	−4.41 (−8.37; −0.45)	−2.70 (−11.30; 5.89)
Household poverty∗Employment precariousness (95% CI)	0.06 (−0.16; 0.28)	0.02 (−0.13; 0.17)	0.02 (−0.13; 0.16)	−0.10 (−0.21; 0.02)	−0.04 (−0.29; 0.21)
Random effects					
Random intercept (Standard error)	5.55 (4.45)	10.16 (7.88)	0.00 (0.00)	13.23 (7.60)	17.63 (18.84)
Residual variance (Standard error)	302.66 (10.40)	352.73 (12.39)	367.40 (11.66)	391.36 (9.14)	428.55 (21.36)
Intraclass correlation coefficient (95% CI)∗∗	0.01 (0.00; 0.07)	0.03 (0.01; 0.12)	0.00 (0.00; 0.04)	0.04 (0.01; 0.10)	0.04 (0.00; 0.23)

*N* Number of participants.

∗ Household poverty was recoded: those who experience no difficulty versus those who experience difficulty.

∗∗ ICC of constant-only model.

**Table 11:** Final models for the association between EP and mental health amongst men and women moderated by subjective household poverty. Adjusted for age, education, having a partner and having children under age 18.

	Total sample (*N* = 18,566)	Total men (N = 8778)	Total women (N = 9788)
Fixed effects			
Intercept (95% CI)	90.57 (87.89; 93.26)	92.52 (88.92; 96.12)	87.76 (84.14; 91.37)
Employment precariousness (95% CI)	−0.40 (−0.46; −0.34)	−0.40 (−0.47; −0.33)	−0.39 (−0.45; −0.33)
Household poverty (95% CI)	−4.06 (−6.72; −1.40)	−2.45 (−6.30; 1.40)	−4.84 (−8.50; −1.18)
Household poverty*Employment precariousness (95% CI)	−0.08 (−0.15; −0.01)	−0.09 (−0.19; 0.01)	−0.08 (−0.17; 0.02)
Random effects			
Random intercept (Standard error)	1.96 (1.75)	2.90 (2.87)	1.42 (1.62)
Random slope (Standard error)	0.00 (0.00)	0.00 (0.00)	0.00 (0.00)
Residual variance (Standard error)	364.11 (3.78)	341.59 (5.16)	382.52 (5.47)

*N* Number of participants.

**Fig. 4:**
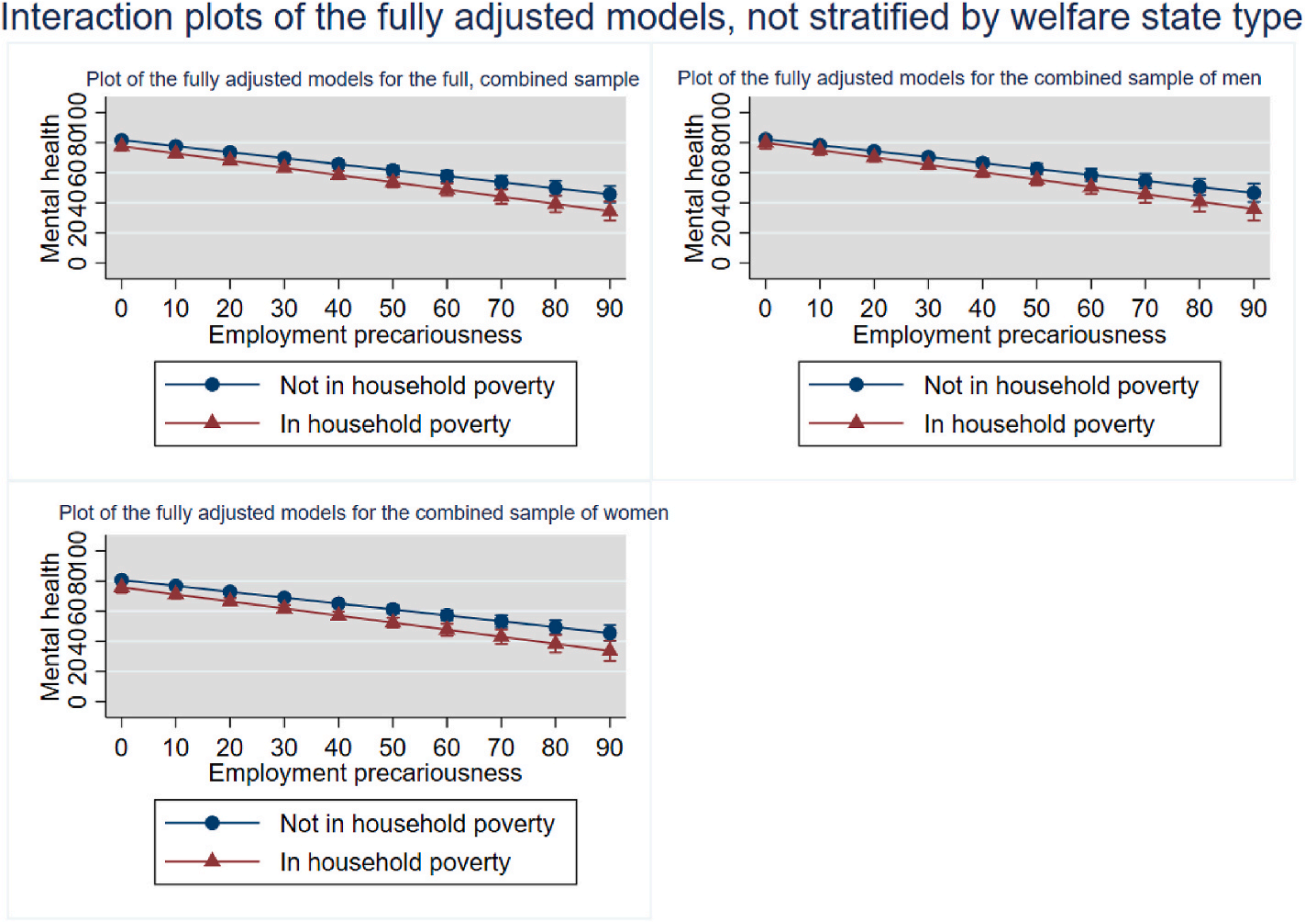
Interaction plots of the association between EP and mental health among the full sample, stratified by subjective household poverty for men and women, separately.

**Fig. 5:**
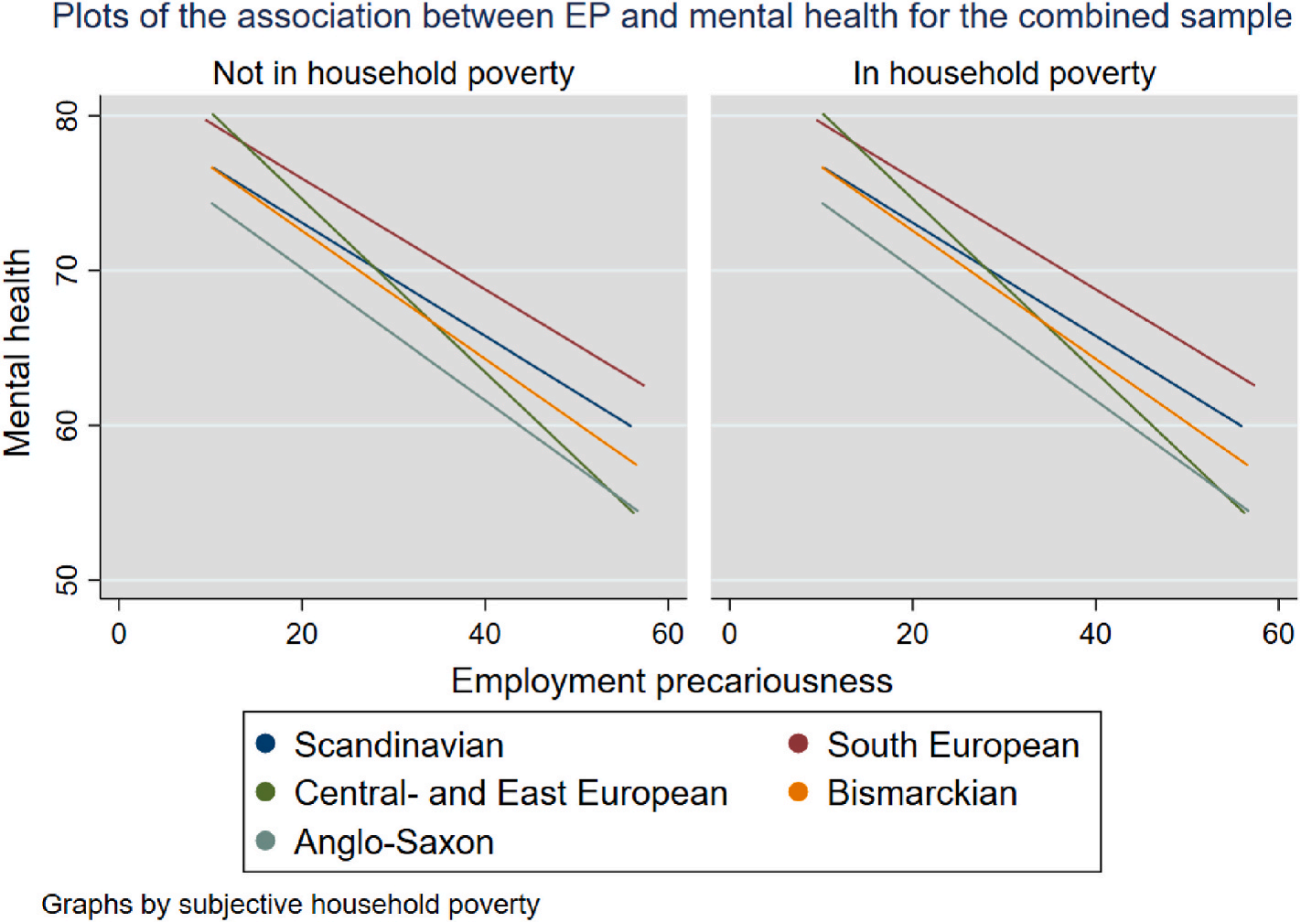
Plots of the association between EP and mental health amongst participants living in household poverty and participants not living in household poverty for both the full, combined sample of men and women.

**Fig. 6:**
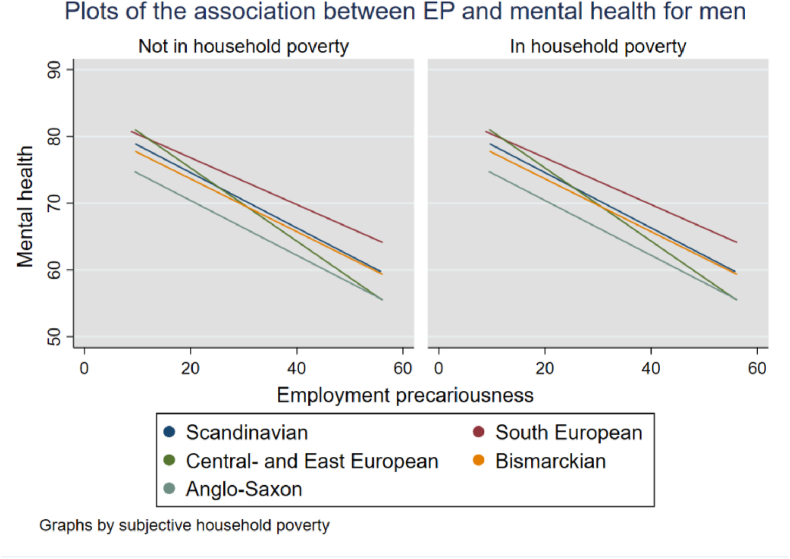
Plots of the association between EP and mental health amongst participants living in household poverty and participants not living in household poverty for both the full sample of men.

**Fig. 7:**
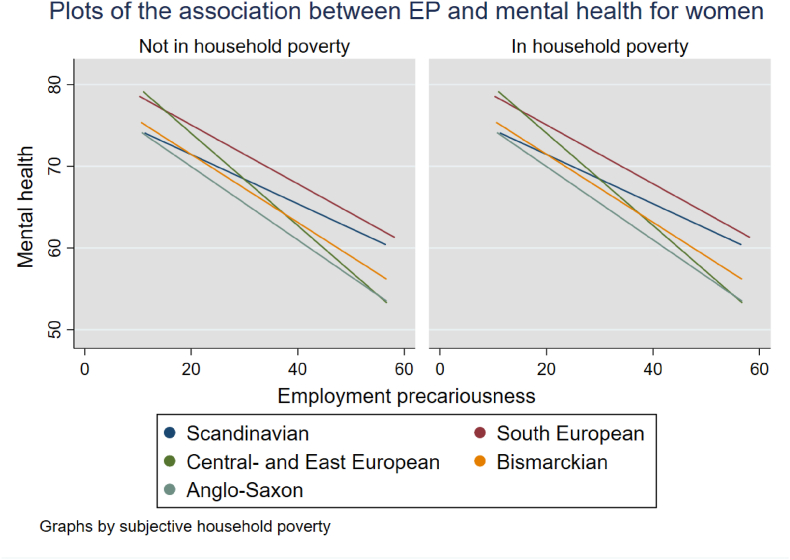
Plots of the association between EP and mental health amongst participants living in household poverty and participants not living in household poverty for both the full sample of women.

## Data Availability

Data from the European Working Conditions Survey 2015 are available from http://discover.ukdataservice.ac.uk.
